# International collaboration in critical care research: a 5-year bibliometric analysis of disparities between critical care studies in developed in high- and middle-/low-income countries

**DOI:** 10.62675/2965-2774.20260420

**Published:** 2026-03-03

**Authors:** Renato Daltro-Oliveira, Victor Hugo Ferreira, Amanda Quintairos, Filipe Sousa Amado, Laura Inez de Oliveira Santos, Jorge Ibrain Figueira Salluh, Antonio Paulo Nassar

**Affiliations:** 1 A. C. Camargo Cancer Center Fundação Antonio Prudente São Paulo SP Brazil A. C. Camargo Cancer Center, Fundação Antonio Prudente - São Paulo (SP), Brazil.; 2 Instituto D’Or de Ensino e Pesquisa Department of Critical Care and Postgraduate Program in Translational Medicine Rio de Janeiro RJ Brazil Department of Critical Care and Postgraduate Program in Translational Medicine, Instituto D’Or de Ensino e Pesquisa - Rio de Janeiro (RJ), Brazil.

## INTRODUCTION

Inequality in scientific production is a persistent global health issue.^(1)^ In critical care, high-income countries (HICs) accounted for up to 90% of published papers between 2018 and 2022.^(2,3)^ This concentration may lead to a mismatch between the research focus and the critical illness burden, which disproportionately affects middle and low-income countries (M/LICs).^(4)^ To assess the extent of global collaboration, we conducted a bibliometric analysis of manuscripts published in intensive care journals.

## METHODS

This is a secondary analysis of a bibliometric study in which we assessed original research studies on adult critically ill patients published from 2018 to 2022 in the 10 highest-impact-factor journals exclusively dedicated to critical care,^(2)^ as reported by Clarivate Analytics.^(5)^ We classified studies as conducted in HICs or M/LICs based on the World Bank country income classification of the corresponding author.^(6)^

Our primary aim was to compare the proportion of multinational studies (i.e., those conducted in more than one country) between HIC- and M/LIC-led studies. Our secondary aims were to compare study designs (randomized controlled trials [RCTs] *versus* observational studies), the proportion of funded studies, the number of patients included, participating centers, and participating countries. Finally, we compared the proportions of HIC-led studies that included M/LICs and of M/LIC-led studies that included HIC countries. We presented categorical variables as absolute counts and proportions and compared them using the Chi-squared or Fisher's exact test. We presented numeric variables as medians and interquartile ranges and compared them with the Mann-Whitney test.

## RESULTS

**Figure 1:**
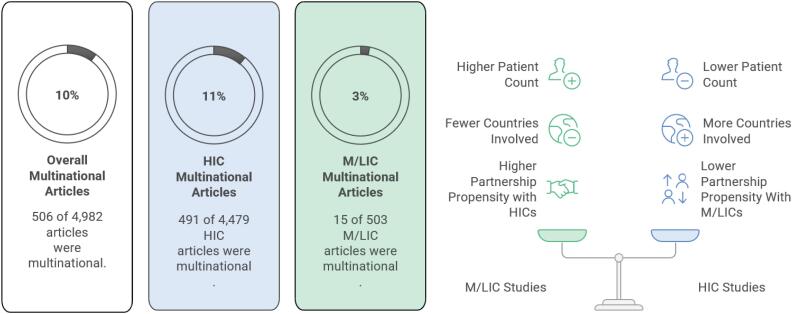
Disparity in multinational critical care research between high-income countries and middle/low-income countries.

Overall, 506 of 4,982 studies (10%) were multinational - 491 of 4,479 from HICs (11%) and 15 of 503 from M/LICs (3%; p < 0.01). There were RCTs only among the HIC-led studies (77 [15.7%], *versus* 0 [05]; p = 0.14), and the proportion of funded studies was similar across income levels (343 [70%] *versus* 11 [73%]; p = 0.99). High-income countries-led studies included fewer patients (694 [233.5 - 2,031.5] *versus* 2179 [473 - 4,707.5]; p = 0.18), but more participating centers (24 [8-70] *versus* 18 [14 - 31]; p = 0.44) and participating countries than L/MIC-led studies (3 [2 - 8] *versus* 2 [2 - 4]; p = 0.12). Notably, fewer HIC-led studies included L/MIC countries than L/MIC-led studies included HIC countries (138 [28.1%] *versus* 9 [60%]; p = 0.02) (Figure 1).

HIC - high-income countries; M/LIC - middle/low-income countries.

## DISCUSSION

These findings underscore that the inequality in research production extends beyond the absolute number of publications, as it also affects the network architecture of scientific collaboration. HIC-led studies are far more likely to involve multinational partnerships. At the same time, LMICs participate mostly as collaborators rather than leaders, potentially reflecting structural barriers and limited resources for international research networking in these settings.^(2,3,7)^

The observation that M/LIC-led multinational studies enrolled more patients and centers, despite involving fewer countries, suggests that the few collaborative projects undertaken by M/LICs tend to be large-scale, resource-intensive endeavors. The fact that M/LIC studies were more likely to partner with HICs than the opposite indicates an asymmetrical collaborative dynamic. While M/LIC researchers proactively seek international partnerships, HIC-led research appears less frequently to engage M/LIC partners.

These asymmetries are concerning because HIC-centric research, despite being more frequent and better funded, may have limitations in knowledge translation and, therefore, lack global relevance due to differences in critical illness epidemiology, health system infrastructure, and practice in M/LICs.^(8)^ To ensure the generalizability of critical care evidence, global alliances are fundamental. Addressing this inequality requires more substantial editorial commitment to inclusivity, greater funding opportunities for M/LIC researchers, and a shift towards equitable collaboration models that prioritize M/LIC-specific research questions. Future research should investigate the outcomes and impact of these asymmetrical collaborations.

## Data Availability

After publication the data will be available on demand to author.
